# Protein signature-based estimation of metagenomic abundances including all domains of life and viruses

**DOI:** 10.1093/bioinformatics/btt077

**Published:** 2013-02-15

**Authors:** Heiner Klingenberg, Kathrin Petra Aßhauer, Thomas Lingner, Peter Meinicke

**Affiliations:** Department of Bioinformatics, Institute for Microbiology and Genetics, University of Göttingen, 37077 Göttingen, Germany

## Abstract

**Motivation:** Metagenome analysis requires tools that can estimate the taxonomic abundances in anonymous sequence data over the whole range of biological entities. Because there is usually no prior knowledge about the data composition, not only all domains of life but also viruses have to be included in taxonomic profiling. Such a full-range approach, however, is difficult to realize owing to the limited coverage of available reference data. In particular, archaea and viruses are generally not well represented by current genome databases.

**Results:** We introduce a novel approach to taxonomic profiling of metagenomes that is based on mixture model analysis of protein signatures. Our results on simulated and real data reveal the difficulties of the existing methods when measuring achaeal or viral abundances and show the overall good profiling performance of the protein-based mixture model. As an application example, we provide a large-scale analysis of data from the Human Microbiome Project. This demonstrates the utility of our method as a first instance profiling tool for a fast estimate of the community structure.

**Availability:** http://gobics.de/TaxyPro.

**Contact:** pmeinic@gwdg.de

**Supplementary information:** Supplementary Material is available at *Bioinformatics* online.

## 1 INTRODUCTION

Metagenomics has significantly enhanced the exploration of the biological diversity on our planet. Shotgun sequencing of environmental DNA has provided a wealth of data for the analysis of the taxonomic and functional composition of a broad range of microbial communities. To make sense of the vast amount of sequences, novel tools had to be developed that could cope with the anonymous and fragmented nature of metagenomic data. In particular, to answer the classical ‘who is there?’ question, several specific problems have to be addressed. The short length of sequence fragments, the insufficient phylogenetic coverage of current genome databases and the computational expense of the underlying algorithms are still limiting factors in taxonomic profiling of metagenomes today. Because we usually have no a priori knowledge about the composition of a metagenome, a taxonomic profiling method, in principle, must be able to cover all three domains of life. To encompass the whole spectrum of possible biological sources, besides bacteria, archaea and eukaryota, also viral entities have to be considered. A major problem of such a full-range taxonomic analysis arises from the limited coverage of genome databases that provide the required reference data for the characterization of novel sequences. In particular, archaea and viruses are generally not well-represented by current database genomes, making it difficult to obtain realistic estimates of the corresponding abundances.

The existing methods for taxonomic profiling can be divided into homology-based and model-based approaches. Among the homology-based approaches, most methods rely on a BLAST (Altschul *et al.*, 1990) similarity search of metagenomic sequences against a genomic reference database (e.g Huson *et al*., 2007; Krause *et al.*, 2008; Meyer *et al.*, 2008). In this case, the phylogenetic classification of sequences is derived from the taxonomic labels of those reference sequences with a significant similarity. This, in principle, enables the measurement of the proportions of bacterial, archaeal, eukaryotic and viral sequences. To reduce the computational cost of the homology search step, the analysis can be restricted to certain signature (Segata *et al.*, 2012b) or marker (Liu *et al.*, 2011) genes. However, in the latter case, the estimation of viral abundances is complicated by the fact that there are no universal marker genes for viruses.

Model-based approaches build a taxon-dependent, in most cases organism-specific, feature representation of reference genomes that is used for the analysis of metagenomic sequences. Currently, all methods rely on features derived from the oligonucleotide frequencies of the taxonomically labeled reference genomes (e.g. Brady and Salzberg, 2009; Rosen *et al*., 2011), which are therefore often termed as composition-based methods. While in the case of homology-based methods the computational cost for taxonomic profiling increases with the number of reference sequences, for model-based approaches, the cost just increases with the number of represented taxons. A good profiling accuracy of model-based methods has been reported for microbial metagenomes that mainly comprise bacterial DNA (e.g. Diaz *et al*., 2009; Meinicke *et al*., 2011; Parks *et al*., 2011; Rosen *et al*., 2011). Recently, also the profiling of metagenomes with a substantial fraction of eukaryotic DNA has been addressed and promising results have been reported (Brady and Salzberg, 2011). However, to our knowledge, there is no systematic study that investigates the ability of model-based approaches to measure the fraction of viral DNA. Conceptually, it is not clear whether unique viral oligonucleotide signatures exist that make them generally distinguishable from microbial signatures. Besides a possible host adaptation, there is no reason why viruses should exhibit a typical codon usage or oligonucleotide profile. The only model-based tool that explicitly includes viral reference data is NBC (Rosen and Lim, 2012). However, as outlined by the authors, the NBC tool should not be used with mixed reference databases of, for example, viral and prokaryotic models because of a preference of NBC estimates for longer genomes. Thus, NBC virus models are not meant to supplement the microbial models for full-range taxonomic profiling and should therefore only be applied to pure virus data where no microbial contamination is anticipated.

We here present the ‘Taxy-Pro’ method as the first model-based taxonomic profiling approach that uses protein signatures instead of the commonly used oligonucleotide signatures. The features of these protein signatures comprise the frequencies of protein domain families according to the Pfam database (Finn *et al.*, 2010). Because it does not make sense to estimate the protein signature of a single short sequence, we do not perform a classification of metagenomic reads by means of these protein features. Instead we make use of a mixture model for taxonomic profiling that has been introduced for oligonucleotide-based profiling of metagenomes (Meinicke *et al.*, 2011). For mixture modeling of protein signatures, we aim to reconstruct the overall Pfam domain frequencies of a metagenome by a linear combination of genomic reference signatures. Pfam domain hits have also been used as a basis for taxonomic profiling by the CARMA (Krause *et al.*, 2008) and TreePhyler (Schreiber *et al.*, 2010) tools. However, these tools do not analyze the overall domain frequencies, but perform a computationally expensive phylogenetic classification of each sequence with a Pfam hit based on a multiple alignment with the Pfam reference sequences.

In the evaluation of Taxy-Pro, we show that the protein-based mixture model provides important advantages when compared with the oligonucleotide-based model. We observed a higher accuracy of Taxy-Pro in the quantification of archaeal DNA, where the narrow spectrum of available database genomes requires a good generalization of the profiling method. In addition, we demonstrate that protein-based models can be built from viral metagenomes to obtain realistic estimates of the virus fraction. Our results indicate a strikingly higher sensitivity of the extended Taxy-Pro mixture model when compared with homology-based classification methods merely based on genomic reference data. Using the domain frequencies as obtained from the CoMet web server (Lingner *et al.*, 2011), computation is about three orders of magnitude faster than a speed-optimized BLAST analysis that includes viral metagenome sequences as reference data.

Because of this computational efficiency, we were able to perform a large-scale analysis of sequence data from the Human Microbiome Project (HMP, Peterson *et al*., 2009) without using a computer cluster or special hardware. In agreement with previous HMP studies (e.g. Huttenhower *et al*., 2012), we found that archaea are not among the abundant organisms in any of the samples. However, we observed a significant eukaryotic fraction in some samples, which has not been reported in the original studies.

## 2 SYSTEM AND METHODS

Our novel taxonomic profiling approach ‘Taxy-Pro’ is based on mixture modeling of the protein domain frequencies in a metagenome as described in the following. We argue that the use of functional reference profiles as mixture model components provides a powerful framework to cope with the inherent underrepresentation of certain biological entities in current genome databases. This problem is especially evident in the case of viruses, and, in fact, it seems not possible to accurately estimate the fraction of viral DNA in metagenomes merely based on genomic reference data. To overcome this limitation, we explored the possibility of including metagenomic reference data to improve the profiling accuracy. While the inclusion in Taxy-Pro is straightforward, a classical BLAST-based pipeline suffers from the computational burden of such an extension. We therefore realized a speed-optimized ‘Combi-BLAST’ method to provide a classical ‘baseline’ approach for comparison with Taxy-Pro (see Supplementary Material).

### 2.1 Protein-based mixture modeling

Our new method for taxonomic profiling combines the detection of protein domains with a mixture model reconstruction of the resulting domain frequencies by means of taxonomically labeled protein reference signatures. Thus, our Taxy-Pro approach comprises two steps: first, we estimate the overall protein domain distribution of a metagenome by relative frequencies of Pfam hits using the CoMet domain detection engine. The resulting profiles comprise the relative frequencies of 12 621 protein domain families according to release 24 of the Pfam database. Then we approximate the Pfam profile vector **y** of a metagenome by linear combination of the precalculated protein reference signatures 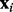 with mixing weights *w_i_* according to
(1)
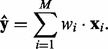

For estimation of the mixture weights, we use the Expectation-Maximization (EM) algorithm (Dempster *et al.*, 1977), whereby 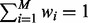 and 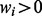. Summing up the mixture weights of all viral reference signatures, we finally obtain an estimate of the virus fraction in a given metagenome. The fraction of bacterial and archaeal DNA as well as the composition on more specific phylogenetic levels was estimated in the same way by summing up the weights of the corresponding reference signatures.

The approximation error of the above mixture model, i.e. the divergence between the original Pfam profile vector **y** and its approximation 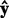, we refer to as the fraction of domain hits unexplained (FDU). The FDU was calculated according to
(2)
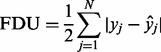

which is half the Manhattan distance between the two profile vectors and therefore ranges between 0 and 1. In terms of the used protein signatures, the FDU measures the fraction of Pfam domain hits that could not be reconstructed by the model, i.e. the fraction that is missing in the reconstructed profile vector 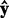 of predicted domain frequencies. Owing to symmetry, there is an equally sized fraction of domain hits that are overpredicted by the model, i.e. that are not present in the actual metagenome profile **y**. Together, these two fractions make up the complete Manhattan distance according to the overall *L*_1_ approximation error. This error is an important indicator of the reliability of the abundance estimates: the better the model fits the actual distribution of Pfam hits, i.e. the lower the FDU value, the more we can trust the taxonomic profile that directly results from the estimated mixture weights. Note that half the Manhattan distance can also be used to measure the divergence between two taxonomic profiles, whereby the *y*-values represent different relative abundances of phyla. In this context, the distance measure corresponds to the Bray-Curtis dissimilarity, which is widely used in ecology for comparison of two assemblages (Bray and Curtis, 1957).

### 2.2 Reference signatures from viral metagenomes

For the reference signature vector profiles 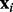, we not only used genomic Pfam profiles of several prokaryotes, eukaryotes and viruses, but we also used a collection of metagenomes for computation of reference vectors. Because the number of available phage genomes, which have a sufficient length for estimation of Pfam profiles, is rather small and variation of viral signatures is large, we use additional reference signatures obtained from a collection of viral metagenomes. In total, Taxy-Pro includes 92 phage genomes and 102 viral metagenomes in addition to 1730 bacterial, 122 archaeal and 50 eukaryotic genomes to obtain 2096 functional reference signatures. Because the estimation of Pfam profiles requires a sufficient amount of DNA sequence, we only use phage genomes with a length >100 kbp for computation of a reference signature.

Depending on the particular treatment, the viral metagenome data in current databases contain a varying amount of microbial contamination. For reasons of accuracy the mixture approach should not be used with viral signatures that arise from sequence data with only a small virus fraction. Therefore, we implemented a selection criterion that requires the reference signatures from viral metagenomes to be well distinguishable from microbial metagenome signatures. Using the Pfam profiles of 260 metagenomes from the CAMERA website (http://camera.calit2.net/, as of April 2010), we trained linear classifiers to discriminate between viral and microbial metagenomes.

A step-wise elimination was applied to reduce the signatures: regularized least squares classifiers on Pfam domain frequencies (Lingner *et al.*, 2010) were optimized by a 5-fold cross-validation to minimize the classification error. The optimization was performed 10 times, each time with a different random partition for the cross-validation. Therefore, each signature was used 10 times as a test example and the one with the highest misclassification rate over the 10 runs was eliminated. The whole procedure was repeated for the reduced set of signatures until no test error occurred during the 10 cross-validation runs. In that way the original number of 151 viral metagenomes was reduced to 102 viral reference signatures that were finally included in the mixture model (see Supplementary Table S3).

In contrast to the oligonucleotide signatures in the original Taxy approach, the Pfam signatures in Taxy-Pro depend on sequence length. To make genomic and metagenomic Pfam profiles compatible, we fragmented the genomic sequences before performing the CoMet domain detection. For that purpose, we used half overlapping fragments with a 400 bp length.

## 3 IMPLEMENTATION

Taxy-Pro is implemented in the MATLAB programming language as an extension of the platform-independent and freely available Taxy toolbox for MATLAB/Octave (http://gobics.de/TaxyPro). In addition, Taxy-Pro has been included in the CoMet web server (http://comet.gobics.de/) to make the method accessible via an easy-to-use interface.

## 4 EXPERIMENTAL EVALUATION

Taxonomic profiling of metagenomes involves two dimensions of resolution: the depth of the analysis indicates the specificity of taxonomic categories along the phylogenetic tree and increases from upper to lower levels. The breadth or range of the analysis indicates the completeness of taxonomic categories on a particular level and increases with the number of branches that are actually included in the estimate. While it may be possible in particular cases, e.g. in metagenomic diagnostics, to measure the taxon abundances on phylogenetically rather specific levels down to genus or even species rank, this would be difficult to achieve for the general case. Without prior knowledge about the true metagenome structure, it is therefore important to first obtain a coarse but reliable high-level estimate that covers the full range of biological entities. Ideally, this estimate includes the fractions of all domains of life and viruses. In our experimental evaluation, we want to point out the difficulties of such a full-range approach and we provide two comparative studies to show the large divergence of results from different tools even on the highest taxonomic level. Finally, we investigate the additional possibility of Taxy-Pro to characterize the uncertainty of a composition estimate by means of the model approximation error.

### 4.1 Measuring archaeal DNA in metagenomes

Microbial genome projects have introduced a strong bias in the databases toward certain kinds of terrestrial bacteria. As a consequence, archaeal organisms are currently underrepresented in genome databases, which complicates the detection of archaeal DNA in metagenomes because taxonomic profiling methods inherently depend on the available reference genomes. To investigate how this underrepresentation affects the composition estimates, we compared method-specific predictions for archaea and bacteria using real metagenomic data and simulated sequencing reads.

The metagenomic dataset we used for comparison of different profiling methods is derived from oil seep metagenome samples as described in (Håvelsrud *et al.*, 2011). For the *Oil Seep* data, a large proportion of archaeal DNA (see Supplementary Material) has been reported in the original study.

**Fig. 1.**
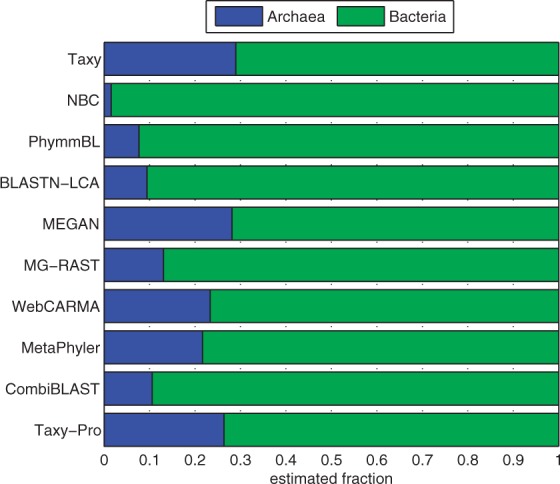
Estimated fraction of archaeal and bacterial DNA for the *Oil Seep* dataset using different tools

To investigate whether this finding could be reproduced with a broad range of methods, we analyzed the results of 11 different tools for taxonomic profiling (see Supplementary Material). For comparison of the composition estimates, we considered only assignments to the two superkingdom categories ‘Archaea’ and ‘Bacteria’ and required the two fractions to yield unit sum. Figure 1 shows the results of our analysis, which reveals that the estimated fraction for archaea varies considerably across different methods. For instance, the oligonucleotide-based NBC method attributed only 1.6% of the classified reads to this domain. On the other extreme, Taxy, MEGAN, Taxy-Pro, WebCARMA and MetaPhyler predicted a relatively large fraction of archaeal DNA (29.0, 28.1, 26.3, 23.4, 21.7%, respectively). The remaining methods produced archaea fraction estimates of ∼10%. This broad spectrum of estimates indicates that the different approaches are obviously affected in different ways by the above-mentioned database bias. We also applied MetaPhlAn (Segata *et al.*, 2012b) to this dataset, but no valid taxonomic assignments could be found with that tool, possibly owing to the low coverage of signature gene hits and the used classification rule (see Supplementary Material).

To further investigate the high degree of disagreement between different approaches, we conducted a simulation study where we used two particular archaeal genomes to generate sequence fragments for taxonomic profiling. For archaea in current databases, the phylogenetic coverage in terms of the number of fully sequenced organisms is rather low for most of the lower rank taxonomic categories. Therefore, it was possible to choose two genomes from rather isolated branches to simulate sequencing reads with a high degree of phylogenetic novelty. To obtain transparent accuracy measurements, we performed two independent tests on species-specific collections of sequences according to the two archaeal organisms. For each collection, we measured the profiling accuracy in terms of the estimated abundances of the true taxonomic categories the organisms belong to. In the ideal case, the estimated fraction for each of the annotated taxons is equal to 1. For a comparative evaluation, we selected five tools for which we could explicitly choose the used reference genomes to provide a strict separation between training and test data. For the sequence classification methods (PhymmBL, MEGAN, SOrt-ITEMS), we only used reads with a valid taxonomic classification for abundance estimation. While Taxy estimates are based on the evaluation of all reads, Taxy-Pro only uses valid Pfam hits for profiling. We included SOrt-ITEMS (Monzoorul Haque *et al.*, 2009) as another protein homology-based tool to investigate the impact of a modified classification rule as compared with the original lowest common ancestor (LCA) rule of MEGAN (Huson *et al.*, 2007).

We used MetaSim (Richter *et al.*, 2008) to simulate reads for the two archaea *Acidilobus saccharovorans 345-15* (As) and *Methanothermus fervidus DSM 2088* (Mf) (see Supplementary Material). Both organisms have been included in NCBI in 2011 and are unique at the rank of order, i.e. there are no overlaps on order level (and below) with the used reference organisms. Also the Pfam release 24 used for CoMet domain detection did not include sequences from these species and correspondingly we excluded all associated hits from the BLAST results in our comparative study. To simulate different read lengths, we generated two test sets with an average fragment length of 250 and 450 bp, respectively.

**Fig. 2.**
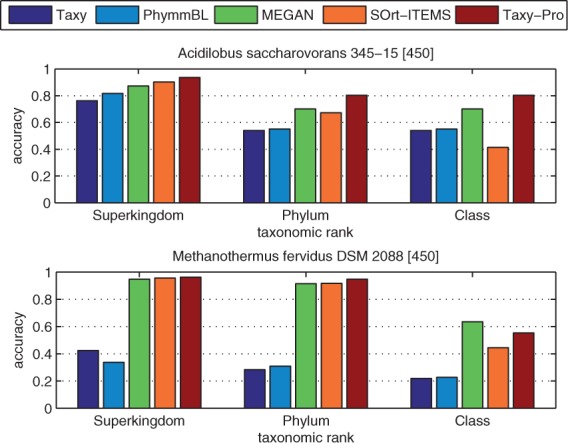
Accuracy of different profiling methods on test datasets from two archaeal genomes using simulated 450 bp reads (see text)

Figure 2 shows the profiling accuracy of different methods in terms of the abundances of the correct taxons on superkingdom, phylum and class level for the 450 bp sequence datasets. In case of MEGAN/SOrt-ITEMS, the percentage of reads with valid taxonomic assignments was 64.9/77.5% for the As data and 80.8/90.5% for Mf sequences. For Taxy-Pro estimates, 45.3% and 65.5% of the reads were used from As and Mf data, respectively. With PhymmBL and Taxy, all reads were used for abundance estimation. On superkingdom level, Taxy-Pro and the two protein homology-based approaches showed good results with nearly 90% and above for both, As and Mf, genomes. In contrast, the olignucleotide-based Taxy and PhymmBL tools broke down to ∼42% and 34% for the Mf genome, indicating a substantially lower generalization capability in that case. As expected, the accuracy of all methods decreases for lower phylogenetic levels. Here, SOrt-ITEMS showed the largest difference from superkingdom to class level at 450 bp read length for both genomes (48.9 and 51.2 percentage points [p.p.] difference, respectively). Comparing the fragment length-induced differences for each method, we found the divergences to be small (<5 p.p.) in general, with only a few exceptions. The maximum deviation we observed on class level for the SOrt-Items tool, which showed a discrepancy of 20.4/10.7 p.p. (As/Mf). The complete results for the simulated 250/450 bp reads are shown in Supplementary Tables S4 and S5.

### 4.2 Measuring viral DNA in metagenomes

Considering the immense diversity and variation of viral genomes, viruses are notoriously underrepresented in genome databases today. This fact also complicates the evaluation of the ability of different taxonomic profiling approaches to predict the fraction of viral DNA in metagenomes. To circumvent the difficulties that arise from the sparse taxonomy and limited coverage of viral genomes in current databases, we avoided the use of simulated data in the evaluation of the profiling accuracy. Instead, we made use of the rich resources in viral metagenomics (e.g. Mokili *et al*., 2012) and chose two viral metagenomes for evaluation of different profiling methods. The *Freshwater* and *Rumen* datasets (see Supplementary Material) have both been obtained using protocols for enrichment of viral DNA. The majority of sequences in these collections are of viral origin although some microbial contamination cannot be excluded. As indicated in Section 1, not all of the before-mentioned tools can actually be used to measure viral DNA in metagenomes. Because of the evidence against oligonucleotide-based approaches, we restricted our comparative analysis to homology-based techniques that are widely used in viral metagenomics.

**Fig. 3.**
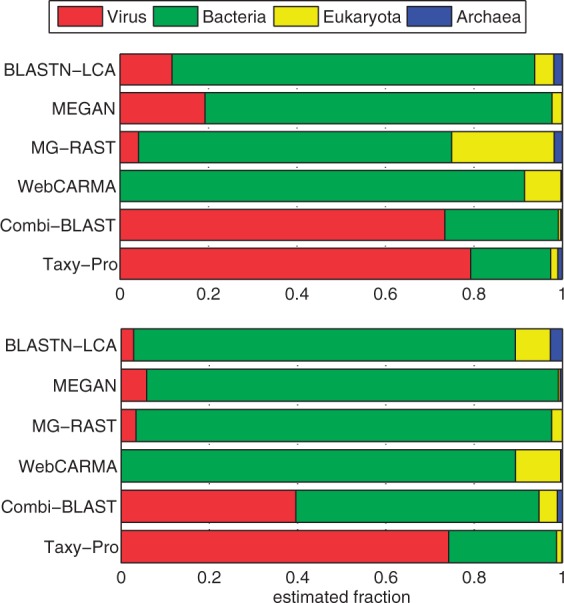
Estimated fraction of viral DNA in the *Freshwater* (top) and *Rumen* (bottom) metagenomes using different profiling tools (see text)

For our analysis, we considered the method-specific proportion estimates on superkingdom level including the fractions of archaea, bacteria, eukaryota and viruses with abundances normalized to yield unit sum. When examining the taxonomic profile as predicted by the different methods, we observed a huge variation in the estimated virus fraction ranging between 0 and 80.4% (Fig. 3). Remarkably, the WebCARMA tool did not classify any reads as viral in any of the two test datasets. Applying the LCA classification rule to the BLASTN output (see Supplementary Material) resulted in virus fraction estimates of 11.8% and 2.8% in *Freshwater* and *Rumen*, respectively. A similar proportion was obtained by the MG-RAST pipeline, which attributed 4.2% and 7.3% to viral categories. Using MEGAN, predicted proportions of 19.2% and 5.8% could be achieved. These were the highest values we obtained with a tool that is based on genomic reference sequences only. Using viral metagenomes as an additional reference, our Combi-BLAST pipeline (see Supplementary Material) estimated 73.4% and 39.6% viral fractions for *Freshwater* and *Rumen*, respectively. The predicted rates were even higher for Taxy-Pro, which produced estimates of 80.4% and 74.0% for these datasets. For both samples, some microbial contamination can actually be expected. In case of the more recent *Rumen* data, the authors identified an unspecified amount of bacterial contamination in the rumen virome sample using 16S rRNA amplification (Berg Miller *et al.*, 2012). This may explain the still significant fraction of bacteria in the Taxy-Pro estimate, which corresponds well with the original study. All methods agreed in predicting only a small fraction of archaea in the viral metagenomes (Fig. 3). Only the WebCARMA and BLASTN-LCA methods detected a relatively high fraction (∼10%) of eukaryotic DNA in the *Rumen* dataset. The relatively low fraction of virus DNA predicted by Combi-BLAST for this dataset indicates a significantly higher sensitivity of the Taxy-Pro approach. An additional evaluation on viral metagenome data that has been obtained with the most recent protocols (Duhaime *et al.*, 2012) showed similiar results with an even higher virus fraction predicted by Taxy-Pro (see Supplementary Figure S1).

### 4.3 Large-scale analysis of Human Microbiome data

The sequence data produced by the HMP (Peterson *et al*., 2009) is the first extensive collection of samples from diverse human body sites with a large number of subjects, allowing for a more precise insight into the composition of the healthy human microbiome. With more than thousand samples, the HMP provides a wealth of data for large-scale comparative studies (e.g. Segata *et al*., 2012a). As a major difference to studies that use particular marker genes for taxonomic profiling (e.g Huttenhower *et al*., 2012), we here analyze all available HMP datasets in terms of their taxonomic composition including all domains of life and viruses. In addition, we investigated the uncertainty of the composition estimates by means of the Taxy-Pro model quality.

#### Model quality

For taxonomic profiling of metagenomes, it is important to characterize the uncertainty of the composition estimates. In a BLAST-based analysis, the proportion of sequences without significant hits can be used as an indicator of uncertainty: the smaller the number of sequences that are actually used to estimate the proportions, the higher the uncertainty of the corresponding estimates. In Taxy-Pro, we have a corresponding measure, namely the amount of sequences without Pfam domain hits, which we refer to as the fraction of sequences unexplained (FSU). Furthermore, with the Taxy-Pro mixture model, it is possible to get an even more comprehensive characterization of the uncertainty by taking into account the approximation error of the model in terms of the FDU (see Section 2).

**Fig. 4.**
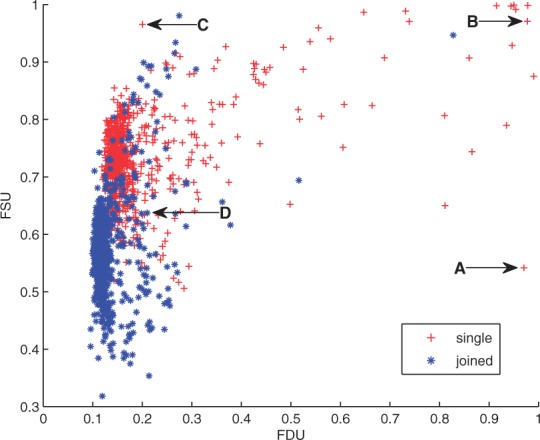
FDU/FSU scatter plot based on 1494 HMP datasets. See text for explanation of markers

To get an overview of the uncertainty in the estimates for the HMP data, we assessed the Taxy-Pro model quality for all datasets in terms of the FSU and FDU values. Figure 4 shows a scatter plot of the two model quality indices for all 1494 samples. Here, the markers associated with the datasets are of different types according to whether they represent ‘joined paired-end’ or ‘singleton’ reads, respectively (see Supplementary Material). Ideally, for reliable composition estimates, a dataset-related marker should be located in the lower left corner of this plot, as a low FDU indicates a well-fitting model, while a low FSU signals a solid statistical basis according to a high fraction of sequences with significant Pfam domain hits. As the stretched shape of the marker distribution in Figure 4 suggests, the FSU values of the HMP datasets exhibit a broader variation than the FDU values. As expected, the data points associated with singleton reads generally show a higher FSU because the effective length of sequences is shorter for the singletons than for the complete paired-end reads. Remarkably, the singleton read data show a considerably higher amount of outliers in the right-hand tail of the FDU distribution. This may be attributed to the fact that the singletons result from a quality check where one part of the paired-end reads did not satisfy the requirements. On average, this may also imply a slightly lower sequence quality of the remaining singletons, as suggested by the horizontal shift of the corresponding (+) cluster in the scatter plot.

An FDU value close to 1 indicates a large approximation error of the Taxy-Pro model for a particular dataset and thus questions the estimates resulting from the model. To investigate possible reasons for an increased FDU, we exemplarily inspected two datasets associated with an extremely high FDU value (markers ‘A’ and ‘B’ in Fig. 4). In one case, the source of uncertainty was quickly identified: the data file associated with marker ‘A’ contains <50 reads, which does not provide a statistical basis for a reliable composition estimate, irrespective of the utilized profiling method. In contrast, the dataset corresponding to marker ‘B’ comprised >60 000 reads. However, an additional BLASTX search against NCBI-nr in this case only showed 323 sequences with significant similarity hits. With such a low coverage of similarity hits also a homology-based profile estimation would be difficult to justify.

On the other side, a low dataset-specific FDU value alone should not be considered a guaranty for a reliable composition estimate. For instance, the sequence data associated with marker ‘C’ in Figure 4 exhibited an extremely low domain hit coverage (FSU: >0.95) paired with a low approximation error (FDU: <0.2). Most reads of this dataset include several unspecified nucleotides within short sequence distance, which prevents the used gene prediction by CoMet from generating a sufficient amount of ORFs longer than the required minimum of 60 bp. In this case, the low fraction of domain hits as compared with the number of sequences suggests to cross-check the resulting composition estimates with a different profiling approach. 

Because of the overall higher FSU and FDU values for the singleton samples as compared with the joined paired-end reads, we excluded the singleton datasets from the subsequent taxonomic profiling analysis with Taxy-Pro. In particular, the many outliers among the singleton samples with rather high FDU values indicate a large quality variation not suitable to provide a consistent statistical basis. From the remaining data, we only excluded one paired-end file with an exceptional FDU of 0.83 to achieve a final number of 749 datasets for the subsequent composition analysis.

#### Composition estimates

Taxonomic profiling of the 749 HMP samples revealed that, as expected, bacteria make up the most abundant fraction in all samples with values ranging between 62.5 and 99.9%. On average, bacteria represented 97.9% (±4.1%) of the estimated abundances. In agreement with the original HMP studies (e.g. Huttenhower *et al*., 2012), we found that archaea are not among the abundant organisms in any of the samples with estimated fractions <1%. The estimated virus fractions were mostly in the range of the prediction error (0.8 ± 0.9%), in almost all cases <5% with only five samples slightly above and with one single sample showing 13.7%. In contrast to the low archaea and virus proportions, we observed a significant eukaryotic fraction in some samples, exceeding 10% in 24 samples with a maximum of 36.3% in one case (on average 1.2 ± 3.8%). This maximum eukaryotic fraction was estimated for a sample from anterior nares (skin, marker ‘D’ in Fig. 4), which we further analyzed with MEGAN. The analysis also resulted in a high predicted fraction of eukaryotic DNA (46.7%), whereby *Malassezia globosa* represented the most frequent Eukaryota species. While members of the genus Malassezia are lipophilic or lipid-dependent yeasts that are part of the normal cutaneous microflora, the excessive abundance of this pathogenic fungus is associated with prevalent skin disorders (Xu *et al.*, 2007).

## 5 DISCUSSION

We have shown that the functional profile of a metagenome in terms of the overall protein domain frequencies can be used to infer its taxonomic composition over the whole range of biological entities. In our evaluation, we demonstrated the difficulties of the existing tools in providing full range estimates that include the proportions of all domains of life and viruses. In particular, a reliable estimation of archaeal and viral fractions constitutes a major challenge for taxonomic profiling tools.

Considering the coverage of current genome databases, we found that it is necessary to include reference data from viral metagenomes to obtain realistic estimates of the virus fraction. Our results indicate that the restriction to genomic reference data in current taxonomic profiling tools leads to systematic underestimation of the virus fraction. For that reason, a large number of taxonomically unassigned reads is sometimes taken as an indirect indicator for a high virus fraction. In general, such kind of negative evidence is not valid and can be completely misleading in metagenomics. Taxy-Pro takes a different approach and performs a taxonomic analysis of the protein signature, which arises from all functionally assigned reads. The Pfam profile may in fact provide positive evidence for viral DNA according to a high similarity with viral reference signatures. Like no other approach, our Taxy-Pro mixture model allows the integration of a large amount of viral reference data from metagenomes in a highly efficient manner. Nevertheless, the limited number and the small range of environments covered by the current data can be expected to introduce a strong bias into the estimation of the viral DNA fraction. Here, our hope is that the rapidly increasing number of sequencing projects will also provide us with a larger number and a broader range of viral metagenomes in the sequence databases.

Also, the quality of the data in terms of a possible contamination is an important aspect. In fact, many of the viral datasets can be expected to contain a significant fraction of microbial DNA. To cope with the contamination problem, we have already shown a possible first solution in terms of a reference selection criterion, which is useful to detect and exclude datasets that cannot be well distinguished from microbial metagenomes on the basis of protein domain signatures. Despite the possible contamination, among all state-of-the-art methods, the Taxy-Pro approach showed by far the most sensitive estimation of viral DNA abundances and therefore our method can significantly contribute to quality control in microbial and viral metagenomics. 

We are aware of the fact that our Taxy-Pro approach relies on the detection of protein domains and therefore is restricted to the coding part of metagenomic DNA. Furthermore, the model quality depends on a sufficient number of Pfam hits, which is difficult to achieve with small datasets or sequence data with large error rates. For instance, a high density of undetermined bases in sequencing reads can substantially decrease the Pfam detection rate (see Section 4.3). Using the domain detection of the CoMet web server, the computation of Taxy-Pro estimates is fast (see Supplementary Table S1) but susceptible to sequencing errors. Metagenomic gene prediction tools that can, in principle, compensate for sequencing errors (Rho *et al.*, 2010) could improve the situation in this case. In addition, HMMER3 (Finn *et al.*, 2011) may be used instead of the faster but less sensitive CoMet engine to further increase the number of Pfam domain hits. We suppose that in principle also approaches based on signature genes, like MetaPhlAn, could be used for abundance estimation across the full taxonomic range. However, the extraction of clade-specific genes that provide a sufficient coverage might be challenging for viruses. In contrast, Taxy-Pro does not require the preselection of specific signature genes but instead considers all Pfam domain frequencies as part of an overall protein signature.

As a novel feature of our approach, Taxy-Pro provides two complementary indices to assess the model quality. The fraction of sequences without Pfam domain hits (FSU) and the FDU can both be used to characterize the uncertainty of a composition estimate. Our large-scale evaluation of datasets from the Human Microbiome Project (HMP) indicates that especially the FDU offers new possibilities for fault detection in metagenomics. Our evaluation on HMP data also shows that Taxy-Pro does not obviate the need for other sequence analysis tools. On the contrary, as a fast pre-screening tool Taxy-Pro should be used in combination with state-of-the-art sequence classification tools to extract a maximum of information from metagenomic data.

## Supplementary Material

Supplementary Data

## References

[btt077-B1] Altschul SF (1990). Basic local alignment search tool. J. Mol. Biol..

[btt077-B2] Berg Miller ME (2012). Phage-bacteria relationships and CRISPR elements revealed by a metagenomic survey of the rumen microbiome. Environ. Microbiol..

[btt077-B3] Brady A, Salzberg S (2011). PhymmBL expanded: confidence scores, custom databases, parallelization and more. Nat. Methods.

[btt077-B4] Brady A, Salzberg SL (2009). Phymm and PhymmBL: metagenomic phylogenetic classification with interpolated Markov models. Nat. Methods.

[btt077-B5] Bray JR, Curtis JT (1957). An ordination of the upland forest communities of southern Wisconsin. Ecol. Monogr..

[btt077-B6] Dempster AP (1977). Maximum likelihood from incomplete data via the EM algorithm. J. R. Stat. Soc. Series B Stat. Methodol..

[btt077-B7] Diaz NN (2009). TACOA: taxonomic classification of environmental genomic fragments using a kernelized nearest neighbor approach. BMC Bioinformatics.

[btt077-B8] Duhaime MB (2012). Towards quantitative metagenomics of wild viruses and other ultra-low concentration DNA samples: a rigorous assessment and optimization of the linker amplification method. Environ. Microbiol..

[btt077-B9] Finn RD (2010). The Pfam protein families database. Nucleic Acids Res..

[btt077-B10] Finn RD (2011). HMMER web server: interactive sequence similarity searching. Nucleic Acids Res..

[btt077-B11] Håvelsrud OE (2011). A metagenomic study of methanotrophic microorganisms in Coal Oil Point seep sediments. BMC Microbiol..

[btt077-B12] Huson DH (2007). MEGAN analysis of metagenomic data. Genome Res..

[btt077-B13] Huttenhower C (2012). Structure, function and diversity of the healthy human microbiome. Nature.

[btt077-B14] Krause L (2008). Phylogenetic classification of short environmental DNA fragments. Nucleic Acids Res..

[btt077-B15] Lingner T (2010). Predicting phenotypic traits of prokaryotes from protein domain frequencies. BMC Bioinformatics.

[btt077-B16] Lingner T (2011). CoMet–a web server for comparative functional profiling of metagenomes. Nucleic Acids Res..

[btt077-B17] Liu B (2011). Accurate and fast estimation of taxonomic profiles from metagenomic shotgun sequences. BMC Genomics.

[btt077-B18] Meinicke P (2011). Mixture models for analysis of the taxonomic composition of metagenomes. Bioinformatics.

[btt077-B19] Meyer F (2008). The metagenomics RAST server - a public resource for the automatic phylogenetic and functional analysis of metagenomes. BMC Bioinformatics.

[btt077-B20] Mokili JL (2012). Metagenomics and future perspectives in virus discovery. Curr. Opin. Virol..

[btt077-B21] Monzoorul Haque M (2009). SOrt-ITEMS: sequence orthology based approach for improved taxonomic estimation of metagenomic sequences. Bioinformatics.

[btt077-B22] Parks DH (2011). Classifying short genomic fragments from novel lineages using composition and homology. BMC Bioinformatics.

[btt077-B23] Peterson J (2009). The NIH human microbiome project. Genome Res..

[btt077-B24] Rho M (2010). FragGeneScan: predicting genes in short and error-prone reads. Nucleic Acids Res..

[btt077-B25] Richter DC (2008). MetaSim: a sequencing simulator for genomics and metagenomics. PLoS ONE.

[btt077-B26] Rosen GL, Lim TY (2012). NBC update: the addition of viral and fungal databases to the Naïve Bayes classification tool. BMC Res. Notes.

[btt077-B27] Rosen GL (2011). NBC: the Naïve Bayes Classification tool webserver for taxonomic classification of metagenomic reads. Bioinformatics.

[btt077-B28] Schreiber F (2010). Treephyler: fast taxonomic profiling of metagenomes. Bioinformatics.

[btt077-B29] Segata N (2012a). Composition of the adult digestive tract bacterial microbiome based on seven mouth surfaces, tonsils, throat and stool samples. Genome Biol..

[btt077-B30] Segata N (2012b). Metagenomic microbial community profiling using unique clade-specific marker genes. Nat. Methods.

[btt077-B31] Xu J (2007). Dandruff-associated Malassezia genomes reveal convergent and divergent virulence traits shared with plant and human fungal pathogens. Proc. Natl Acad. Sci. U S A.

