# Supplementary material for: Protein signature-based estimation of metagenomic abundances including all domains of life and viruses
Source: Bioinformatics. 2013 Feb 15;29(8):973–80. doi: 10.1093/bioinformatics/btt077 (PMC3624802; doi:10.1093/bioinformatics/btt077)
Supplement: Supplementary Data [file supp_29_8_973__index.html]

Protein signature-based estimation of metagenomic abundances including all domains of life and viruses — Protein signature-based estimation of metagenomic abundances including all domains of life and viruses — Supplementary Data 

# Protein signature-based estimation of metagenomic abundances including all domains of life and viruses

## Supplementary Data

files

**Files in this Data Supplement:**

- Supplementary Data - ps file
